# ﻿*Maxillariaanacatalinaportillae* (Orchidaceae, Maxillariinae), a new remarkable species from Ecuador

**DOI:** 10.3897/phytokeys.190.77918

**Published:** 2022-02-17

**Authors:** Monika M. Lipińska, Natalia Olędrzyńska, Alex Portilla, Dorota Łuszczek, Aidar A. Sumbembayev, Dariusz L. Szlachetko

**Affiliations:** 1 Department of Plant Taxonomy and Nature Conservation, Faculty of Biology, University of Gdańsk, Wita Stwosza 59, 80–308 Gdańsk, Poland; 2 Foundation Polish Orchid Association, 81–825 Sopot, Poland; 3 Géneros Ecuatorianos Ecuagenera Cia. Ltda., Km. 2 1/2 Vía a Cuenca Sector Llampasay, PO Box 01.01.1110 Cuenca, Ecuador; 4 Laboratory of Electron Microscopy, Faculty of Biology, University of Gdańsk, Wita Stwosza 59, 80–308 Gdańsk, Poland; 5 Altai Botanical Garden, Ridder, Kazakhstan; 6 Al-FarabiKazakh National University, Almaty, Kazakhstan

**Keywords:** Ecuador, Neotropics, orchids, phylogeny, pseudopollen, SEM

## Abstract

Neotropical genus *Maxillaria* Ruiz & Pav. belongs to one of the most diverse and species-rich groups of orchids. Several of its representatives are popular, horticultural plants with large and showy flowers, often nicely fragranced. It is not uncommon that some distinctly colored individuals are introduced to the commercial market under names of similar, more or less related species, as informal varieties or color forms, largely causing confusion. While investigating the diversity of *Maxillaria* in Ecuador, we have encountered plants that were commercially referred to as *M.sanderianaxanthina*. In the course of conducted morphological and micromorphological analyses, we concluded that it is a new, separate species and hereby, we describe it as *M.anacatalinaportillae*.

## ﻿Introduction

*Maxillaria* Ruiz & Pav. is one of the most interesting species groups in the orchid family. For many years it has been, and in some way still is quite a controversial genus. The lack of clearly defined boundaries of *Maxillariasensu stricto* resulted in proposing several taxonomic approaches of the subtribe Maxillariinae Benth. over the past few decades. For a long time, it has been suspected that it is an assemblage of taxa, consisting of morphologically disparate groups of species (Whitten et al. 2007). Establishing the exact number of species belonging to the various genera or the subtribe itself is not easy since it depends mainly on the adopted classification system and genus concept. *Maxillaria* covers about 4/5 of species belonging to the subtribe (Senghas 2002). Depending on the applied classification, it counts from approximately 420 (Dressler 1993), through 634 (Schuiteman and Chase 2015) to 750 species (Senghas 2002).

**Figure 1.**
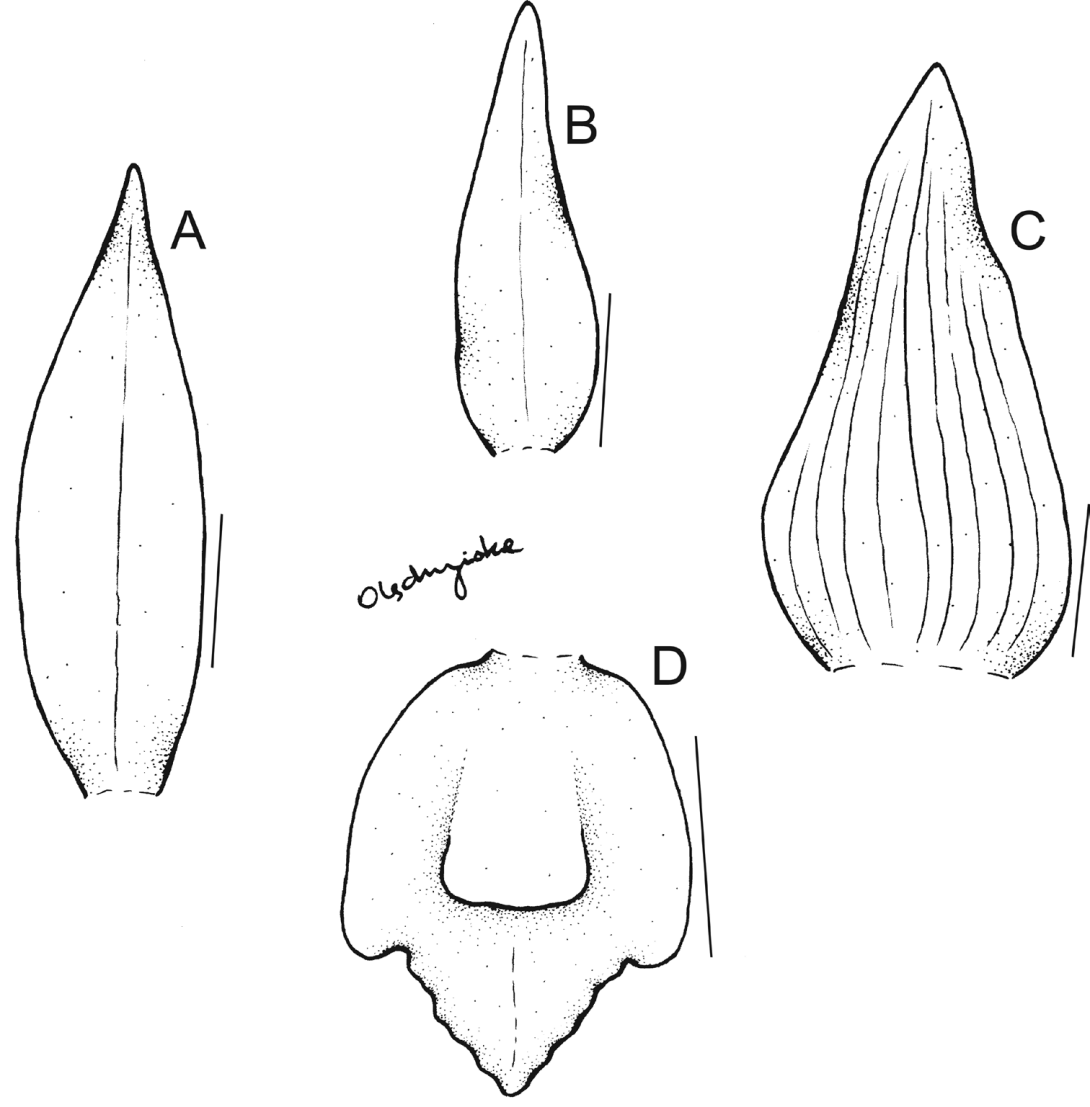
Drawing of the flower parts of *Maxillariagrandiflora***A** dorsal sepal **B** petal **C** lateral sepal **D** lip. Scale bars: 10 mm. Redrawn from the type by N. Olędrzyńska.

One of the most spectacular groups of species within the genus is often referred to as ‘*Maxillariagrandiflora*-complex’ or also alliance/group (Dodson 1997), it includes species such as *M.platypetala* Ruiz & Pav., *M.molitor* Rchb. f., or M.sanderiana Rchb. f. ex Sander. Their common feature is the size of flowers – generally large and showy, sometimes also brightly colored. The main reason for this reference is the supposed similarity to *Maxillariagrandiflora* (Kunth) Lindl. (Christenson 2013). Dodson (1997) characterized this group as caespitose plants with unifoliate pseudobulbs and foliaceous leaf sheaths, petiolate leaf, large flowers (5–12 cm in diameter), and broad and blunt sepals and petals. Christenson (2013) pointed out, however, that the type specimen of *M.grandiflora* itself is bifoliate and several other species included by Dodson, do not fulfill the morphological criteria presented above (e.g. *M.napoensis* Dodson, *M.batemanii* Poepp. & Endl). Indeed, for many years there have been some ambiguities about the type of *M.grandiflora* (Fig. 1) and its locality, however, they have been clarified by Blanco and Stauffer in 2011. Detailed analysis of the collection time, numbers and travel route conducted by them, have led to a conclusion that the type locality of *Maxillariagrandiflora* must be somewhere in the eastern part of La Cruz municipality in the department of Nariño, Colombia at the altitude of 2,067 m. As mentioned above, some questions were raised by Christenson (2002a, b, 2013) about the number of leaves on the pseudobulb of the type specimen. It is essential to clarify that the type collection of *Maxillariagrandiflora* consists of three sheets deposited in the Bonpland herbarium in the Muséum National d’Histoire Naturelle (P) in Paris. In their paper, Blanco and Stauffer (2011) state clearly that in this case we are dealing with a mixed collection. During our visits in P herbarium, we have examined this specimen and we agree with Blanco and Stauffer that it consist of plant parts that belong to at least two different species. As the number of apical leaves per pseudobulb is an important taxonomic feature, the existence of a pseudobulb with two apical leaves as part of the type collection of *M.grandiflora* might have been a source of confusion resulting in Christenson`s belief that this taxon is not a member of the *M.platypetala* Ruiz and Pav. alliance (*sensu*Whitten et al. 2007).

Christenson (2013) pointed out three useful clues for the proper taxonomic identification within *Maxillariagrandiflora*-complex: 1) the orientation of tepals is crucial for understanding the species circumscription (segments reflexed, inflexed, incurved, among others), 2) flower color and lip markings, 3) the shape of the lip apex (transversely terminated, usually undulate, with lobes similar to the ruffles of a petticoat or rigidly held and V-shaped in cross-section). Most of the large-flowered Andean *Maxillaria* species are at times informally referred to as *Maxillariagrandiflora*-complex. This often causes confusion as many of them have little or nothing in common with actual *M.grandiflora* (Kunth) Lindl. The general understanding of these species remains scarce and requires intensified taxonomic investigation. Especially three sister species can be considered as challenging, and these are M.sanderianaRchb.f.ex Sander, *M.grandis* Rchb.f. (sometimes believed to be a synonym of *M.sanderiana*) and *M.wojii* Christenson (often confused with both) (Christenson 2013). However, *M.sanderiana* differs from *M.grandis* Rchb.f. by narrow and strongly undulated petals and from *M.wojii* by significantly larger flowers. *Maxillariasanderiana* and *M.grandis* share the distribution range and occur in Ecuador and Peru. According to Christenson (2013) some may consider them to be two ends of a spectrum of variation over a broad geographic range and in this concept, the name *M.grandis* takes priority and is the correct name for the broadly defined species. The variability within *M.sanderiana* may also suggest that, in fact, it is a complex of species consisting of one or more undescribed taxa. Another theory has been mentioned by Blanco as a comment in Christenson`s monograph (2013). He suggested that *M.wojii* is simply a color variation of *M.grandis* or that both are hybrids of *M.sanderiana* with some other species in the *Maxillariagrandiflora*-complex. This would expend the occurrence range of *M.grandis* as *M.wojii* is known only from Colombia.

During the past few years, we have been working intensely on the classification and especially species delimitations within *Maxillariasensu stricto.* We have investigated herbarium materials deposited in most of the world’s collections, such as AMES, B, C, COL, MA, MO, P, W, W-R to name just a few. During our expeditions to South America, our attention was drawn to the *Maxillariagrandiflora*-complex and many taxonomical ambiguities it brings. We have collected samples from both commercial and hobby growers but also purchased several plants to cultivate them in the greenhouses of the University of Gdańsk. One of them was identified as *M.sanderiana* and was referred to by Ecuagenera as “*xanthina*”. Indeed, according to Christenson (2013), there are two color forms of *M.sanderiana: atropurpurea* (H. Williams) Christenson and *xanthina* Christenson. However, after morphological analysis of the flower, it became clear that we are facing new species and hereby we describe it as a new one. The new entity can be easily distinguished by having flat margins of petals, which are gently angled, callus extending beyond the middle of the lip and form of lip middle lobe, which is broadly cordate or triangular when spread, with fold down margins. It is known from several plants that are available commercially, however, they all originate from single (type) population.

## ﻿Materials and methods

### ﻿Morphological analysis

Flowering plants of the new species were collected on November 11^th^, 2003 in the Carchi province (northern Ecuador). The species was photographed *in situ* and taken to cultivation in the greenhouses of Ecuagenera Cia. Ltda with initial identification as *M.sanderiana ‘xanthina*’. In 2020, Ecuagenera provided plant material consisting of five plants which have been sent to Poland with corresponding CITES certificates. Plants have been cultivated in the greenhouses of the University of Gdańsk (voucher 0148255) and used for the presented analysis. Herbarium specimen were prepared to be used as type material and deposited at UGDA. Particular parts of the flower were dissected, measured, and drawn under stereomicroscope. The line illustration of the new species was prepared from material preserved in Kew Mixture (53% ethanol: 5% formaldehyde: 5% glycerol: 37% water) and digital photos. The new entity has been compared with more than 800 herbarium specimens of other members of *Maxillariagrandiflora*-complex from the following herbaria: AMES, B, C, COL, MA, MO, NY, P, W, W-R, VALLE, QCE, and QCNE. We conducted a careful comparison of the new species with the protologues and type material of all species belonging to the complex, as well as regional floras and checklists such as Dodson and Marmol (1980), Dodson (2002), and Jørgensen and León-Yánez (1999). The conservation status of the new species was evaluated, based on the guidelines of the International Union for Conservation of Nature (IUCN 2019).

### ﻿Phylogenetic analysis

Plant material for molecular analysis has been obtained from plants provided by Ecuagenera and living orchid collection of the University of Gdańsk. Remaining sequences were obtained from NCBI database. The GenBank accession numbers of the used sequences in the study are given in the Appendix 1: Table A1.

Total genomic DNA of three species (*M.anacatalinaportillae*, *M.huebschii*, and *M.melina*) was extracted from ca. 20–25 mg of silica-dried specimens (parts of the leaves), using Sherlock AX Kit (A&A Biotechnology, Poland) and following the original protocol. Two molecular markers were used for phylogenetic reconstruction: nrITS (ITS1-5.8S-ITS2) and plastid *matK.* ITS was amplified using primers 101F and 102R (Douzery et al. 1999), while *matK* using primers 19F (Molvary et al. 2000) and 1326R (Cuénoud et al. 2002).

Polymerase chain reactions (PCR) were carried out in a total volume of 25 µl and containing 12.5 µl of StartWarm HS-PCR Mix (A&A Biotechnology, Poland), 1.0 µl of each primer (10 µM) and 1 µl of DMSO (dimethyl sulfoxide) – only for ITS. The following parameters were implemented: 94 °C – 4 min; (94 °C – 45 s; 52 °C – 45 s; 72 °C – 1 min) × 30; 72 °C – 7 min for ITS and 95 °C – 3 min; (94 °C – 45 s; 52 °C – 45 s; 72 °C – 2 min 30 s) × 33; 72 °C – 7 min for *matK* amplification. PCR products were purified using Wizard SvGel and a PCR Clean Up System (Promega, United States). The sequencing reactions were carried in an external company – Macrogen Europe B.V.

Obtained chromatograms were analyzed and edited using Finch TV (Geospiza). Two separate matrixes (ITS and *matK*) were prepared and then aligned with Mafft software (https://mafft.cbrc.jp/alignment/server/). Minor mistakes were additionally corrected in SeaView v.4. (Gouy et al. 2010).

Molecular substitution model was based on AIC (Akaike information criterion) and calculated with PhyML website (http://www.atgc-montpellier.fr). The GTR+G+I model was selected as the best one for studied matrix.

**Figure 2.**
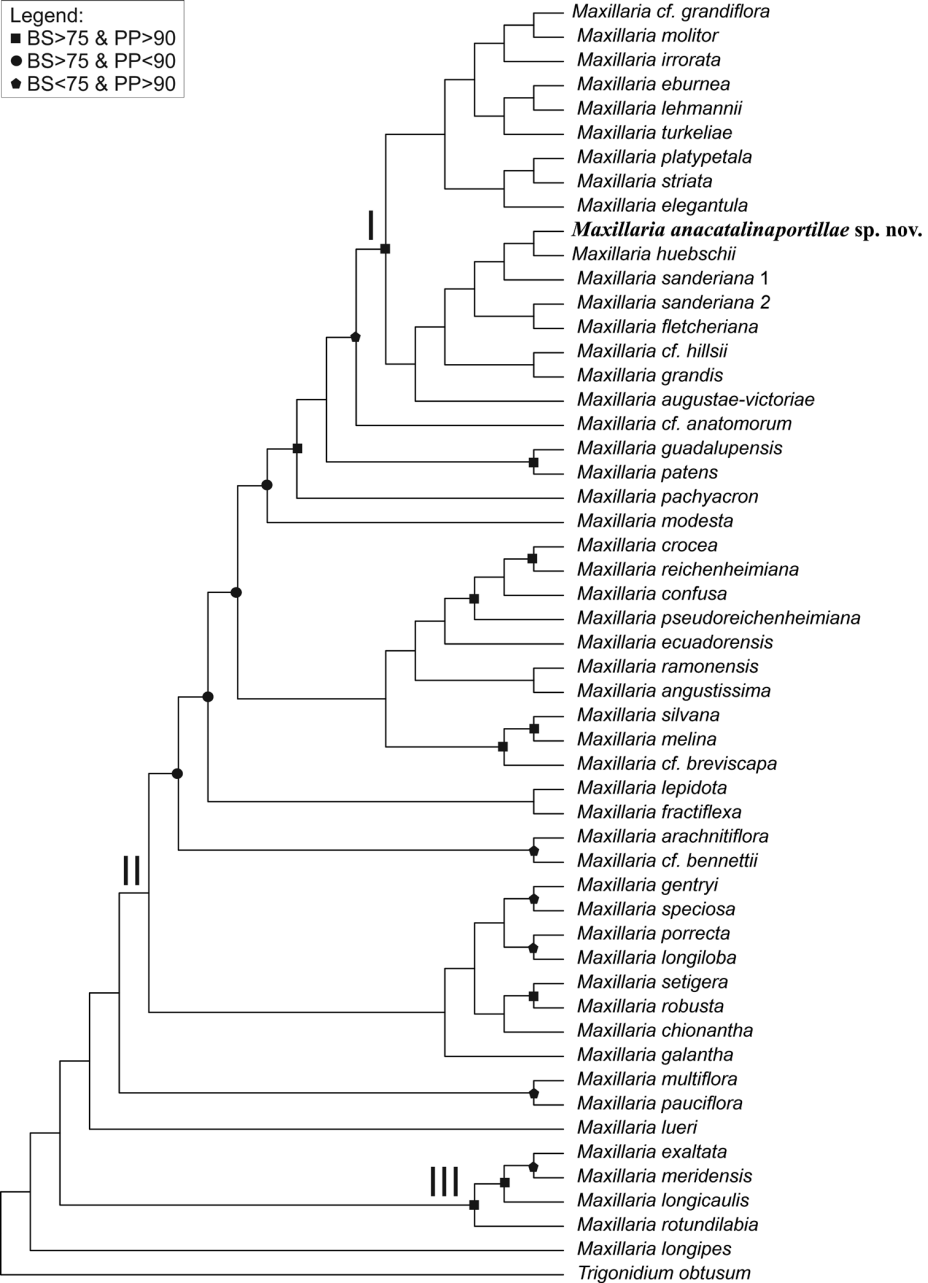
Phylogenetic placement of *Maxillariaanacatalinaportillae*. Maximum clade credibility tree based on combined ITS and *matK* data.

In the first step of phylogenetic tree reconstruction, two separate matrices (for ITS and *matK*) were analyzed using Bayesian Interference and maximum likelihood methods. Finally, due to the low clade support, high polytomy, and no sign of topology conflict, the combined analysis was performed (only the results of this one are shown, Fig. 2). The results of single markers analysis are available upon request.

The Bayesian analyses was performed using Markov chain Monte Carlo (MCMC) in MrBayes 3.2.7a (Huelsenbeck and Ronquist 2001) using CIPRES Science Gateway (Miller et al. 2010). The analysis was performed in two simultaneous runs of four chains for 20,000,000 generations, sampling one tree for each 100, until the average standard deviation of split ranges reached a value < 0.01. TreeAnnotator v. 1.8.1 (Drummond et al. 2012) were used to construct a maximum clade credibility tree, with a burn-in of 25%. The support of the clades was evaluated by the posterior probability (PP).

The Maximum Likelihood analysis was performed using raxmlGUI 2.0 (Edler et al. 2021) using ML + transfer bootstrap expectation + consensus option and 1000 bootsrap (BS) replications.

### ﻿Micromorphological analysis

Samples for the scanning electron microscopy (SEM; voucher no. UGDA.0148255) have been preserved in 2,5% GA and 2,5% PFA in 0,05M cacodylate buffer (pH 7,0). Following dehydration in an ethanol series, they were dried by the critical point method using liquid CO2 and coated with gold. They were observed using a Philips XL-30 scanning electron microscope.

## ﻿Results

### ﻿ Maxillaria anacatalinaportillae

Taxon classificationPlantaeAsparagalesOrchidaceae

Szlach. & Lipińska sp. nov.

2C87298D-5484-5DAF-8506-0EA337623F3B

urn:lsid:ipni.org:names:77255006-1

Figs 3 , 4

Maxillaria anacatalinaportillae Type: ECUADOR. Carchi Province, Maldonado. Alt. 1700 m. 11.04.2003. *A. Portilla* s.n. (Holotype: UGDA-DLSz! – spirit, drawings, photo).

#### Diagnosis.

*M.anacatalinaportillae* appears to be similar to *M.grandis*, *M.sanderiana* and *M.wojii*. The new entity can be easily separated from *M.grandis* Rchb.f. by having flat margins of petals, which are gently angled (vs undulate and strongly recurved petals), longer lip callus extending beyond the middle of the lip (vs lip callus not reaching lip middle point) and form of lip middle lobe, which is broadly cordate or triangular when spread, with fold down margins (vs lip middle lobe oblong-elliptic, with undulate and planar margins). The lip middle lobe of *M.sanderiana* has strongly undulated and planar margins, and petals are shorter than dorsal sepal (vs equal in length in our new species). *Maxillariawojii* can be easily distinguished from all other species mentioned above by unique lip callus, consisting of the main part flanked by pair of subsidiary calli. Lip callus of *M.anacatalinaportillae* is very massive flanked by narrow wings on each side.

#### Description.

**Figure 3.**
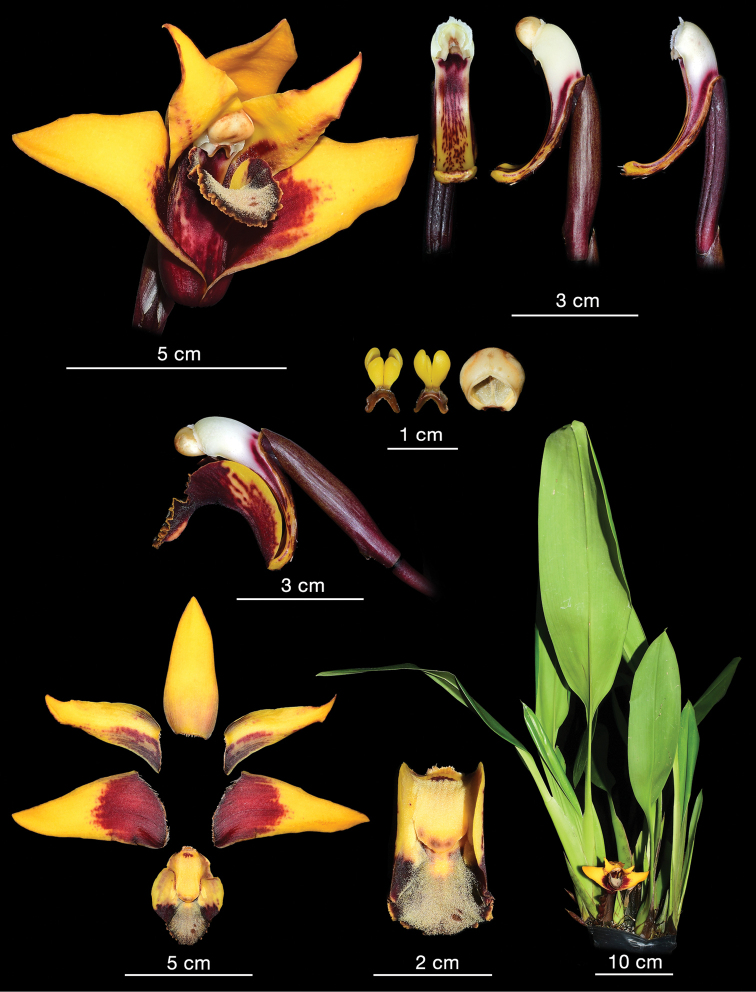
*Maxillariaanacatalinaportillae***A** complete flower **B** column **C** pollinia and anther cap **D** side view of the column and lip **E** perianth parts **F** lip **G** habit. (Phot. Hugo Medina).

Plants caespitose. Pseudobulbs 4–5.5 cm long, 4–4.5 cm wide, ellipsoid to almost orbicular, laterally compressed, unifoliate, supported basally by 1–2 leafy sheaths. Sheaths petiolate; petiole up to 20 cm long, conduplicate, narrow; blade up to 30 cm long and 8.5 cm wide, ligulate to oblong-elliptic, acute to shortly acuminate at apex, basally cuneate. Leaf petiolate; petiole up to 5 cm long, conduplicate; blade up to 33 cm long and 7.5 cm wide, similar in form to sheaths, ligulate to oblong-elliptic, acute to shortly acuminate. Peduncle ca 5–7 cm long, enveloped in 4–5 sheaths, erect, basal, single-flowered; sheaths elliptic-lanceolate, acute, thin, fibrous, brownish. Flowers large and showy, scentless, campanulate, not fully opened, resupinate, sepals red-wine or maroon outside, yellow inside with red-maroon basal part (Fig. 3); petals yellow with red-maroon veins and irregular dots on both sides of the middle vein, lip basal part yellow, callus yellow with dark apical part, middle lobe red-black with grayish suffusion, margins yellow, red-maroon outside, gynostemium yellowish with red-maroon on the ventral surface below stigma, anther yellow. Floral bracts ca 60 mm long, elliptic-lanceolate, greenish-brown with maroon veins. Ovary 30 mm long, glabrous. Tepals thick, fibrous. Dorsal sepal 60–62 mm long, 25–27 mm wide, elliptic-ovate, concave along midvein, apex subobtuse, canaliculated. Petals 60–62 mm long, 23–25 mm wide, oblong-lanceolate to ligulate-lanceolate, falcate at base, apex attenuate, thickened, subobtuse. Lateral sepals 75 mm long, 30–32 mm wide, obliquely oblong triangular, somewhat concave at the base, apex thickened, subobtuse. Lip hinged on the column foot, ca 45 mm long in total, 30 mm wide when spread, very stiff, gently arched, papillate in the apical half, 3-lobed in the apical third, callus very massive reaching beyond the midpoint of the lip, ligulate-ovate, flanked by narrow wing on each side; middle lobe ca 13–15 mm long, 18–20 mm wide, broadly cordate or triangular when spread, concave along midvein, margins crenulate-undulate, fold-down; lateral lobes 30 mm long, oblong-ovate in outline, canaliculated in natural position. SEM analysis revealed the presence of copious moniliform trichomes and pseudopollen grains on the lip surface, mainly middle lobe and callus (Fig. 5). Lip base and lateral lobes were rather smooth, with villiform to obpyriform papillae towards the middle part of the lip (Fig. 5B). These papillae seem to be the early stage in the development of the moniliform trichomes. Gynostemium 23 mm long, column foot 33 mm long, apically upcurved, clinandrium densely glandular.

**Figure 4.**
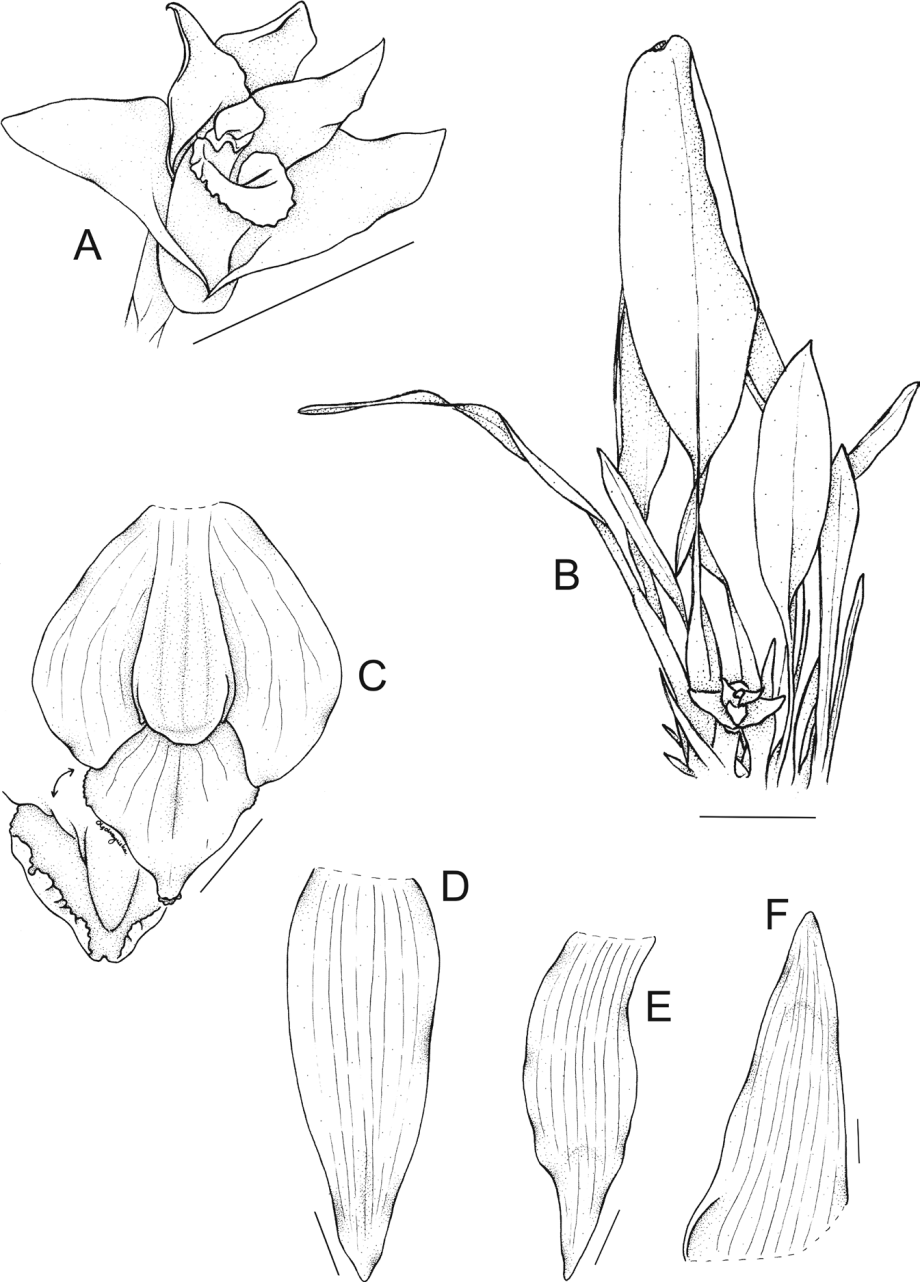
Drawing of the flower parts of *Maxillariaanacatalinaportillae***A** flower **B** general habit **C** lip **D** dorsal sepal **E** petal **F** lateral sepal. Scale bars: 5 cm (**A**); 10 cm (**B**); 10 mm (**C-F**). Drawn by N. Olędrzyńska from the holotype.

**Figure 5.**
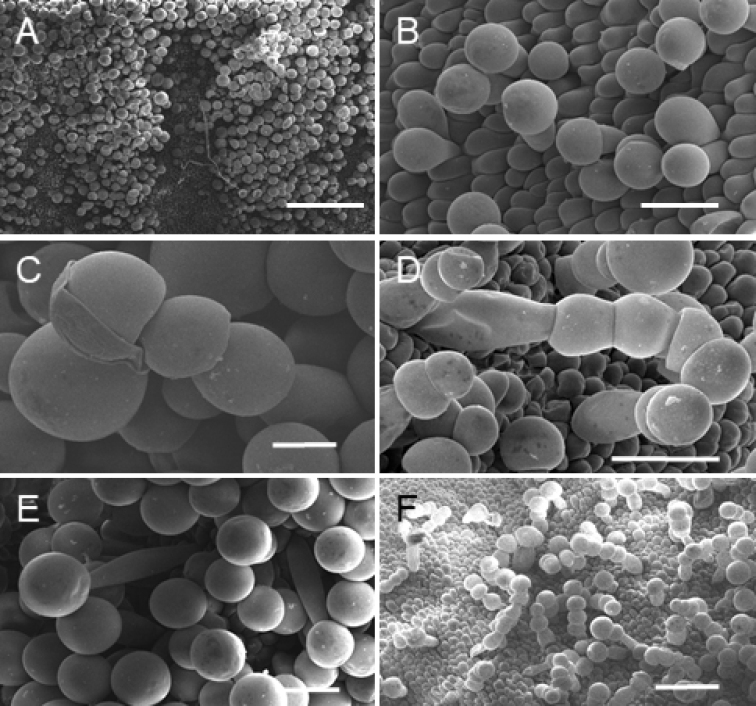
*Maxillariaanacatalinaportillae***A** masses of pseudopollen accumulated on the midlobe **B** conical, villiform, and obpyriform papillae **C** grains of pseudopollen on the single celled trichome **D, E** magnification of moniliform trichomes **F** moniliform trichomes scattered on the lip surface (Phot. D. Łuszczek). Scale bars: 50μm (**A**); 100μm (**B, D, E**); 50μm (**C**); 200μm (**F**).

#### Ecology and distribution.

Grows as an epiphyte in cloud rainforest at altitude of 1700 m asl, known only from the type location.

#### Eponymy.

Name dedicated to Ana Catalina Portilla Schröder – daughter of Alex Portilla, finder of the new entity.

#### Conservation status.

According to the IUCN Red List criteria (IUCN 2019), the new entity should be classified as critically endangered (CR B2ab (iii)), based on the small number of known populations and restricted area of distribution. The province of Carchi in recent years suffered from problems caused by climate change, anthropogenic impact on the environment, and the lack of awareness of natural resources. The change of land use, expansion of the agricultural frontier, population growth, or the opening of new roads are some dynamics that generate pressure on the ecosystems, compromising the ecological processes that take place in them. According to Global Forest Watch, from 2001 to 2020, Carchi lost 8.34 kha of tree cover, equivalent to a 3.7% decrease in tree cover since 2000.

#### Notes.

We know about several living collections in Ecuador that are probably representing the new entity, however, since we were not able to investigate these plants in person, we can only treat them as possible representative specimens. According to A. Hirtz, collections are located in Botanic Garden of Quito, Orquidario Las Juntas (near Gualtal at the south side of the Golondrinas Volcano, owned by Arturo and Esmeralda Mendez), Quinche (near the airport of Quito, collection of Juan Galarza), and Orquidario Casa Dracula in Quinshul (owned by Hector Yela).

#### Phylogeny.

The results are presented on the maximum clade credibility tree obtained from Bayesian analysis. Support of particular clades (PP and additionally BS – from ML analysis) is marked with a square circle or pentagon, according to the legend given on the Fig. 2.

Obtained phylogenetic tree consists of representatives of Maxillariinae, including those recently recognized by some authors (e.g. Szlachetko et al. 2012) genera *Calawaya* (III) and *Pseudocymbidium* (represented by *M.lueri* Dodson = *Pseudocymbidiumlueri* (Dodson) Szlach. and Sitko). *Maxillaria* s. str. (II) seems to be monophyletic, but there is no PP or BS support for this clade. The clade of *Maxillariagrandiflora*-complex is well supported and includes the new species *Maxillatiaanacatalinaportillae*.

## ﻿Discussion

### ﻿Phylogeny

The main purpose of phylogenetic reconstruction in this paper was the placement of the new species, thus the phylogenetic relationship within the Maxillariinae will not be widely discussed here. Our results indicate the affinity of *Maxillariaanacatalinaportillae* to the *Maxillariagrandiflora*-complex. However, the relationships between species within the complex are still unclear. Moreover, some authors (e.g. Whitten et al. 2007) postulated to include the complex into *Maxillariaplatypetala* alliance, due to the relatively low genetic differentiation among the members of these two groups, which may suggest the recent radiation of the species. In our opinion further researches are necessary to make any decision about the taxonomic position of mentioned taxa and to fully resolve the relationships between its species.

### ﻿Morphology

Many representatives of *Maxillariagrandiflora*-complex (*sensu*Christenson 2013) or *M.platypetala* alliance (*sensu*Whitten et al. 2007) are superficially similar to each other but can be easily distinguished when live plants are compared side by side (Blanco and Stauffer 2011 and references their). Some of the distinguishing features, such as color patterns, may disappear or be obscured in dried herbarium specimens; especially flowers tend to become dark brown to almost black when dry, regardless of their original color (Blanco and Stauffer 2011).

**Figure 6.**
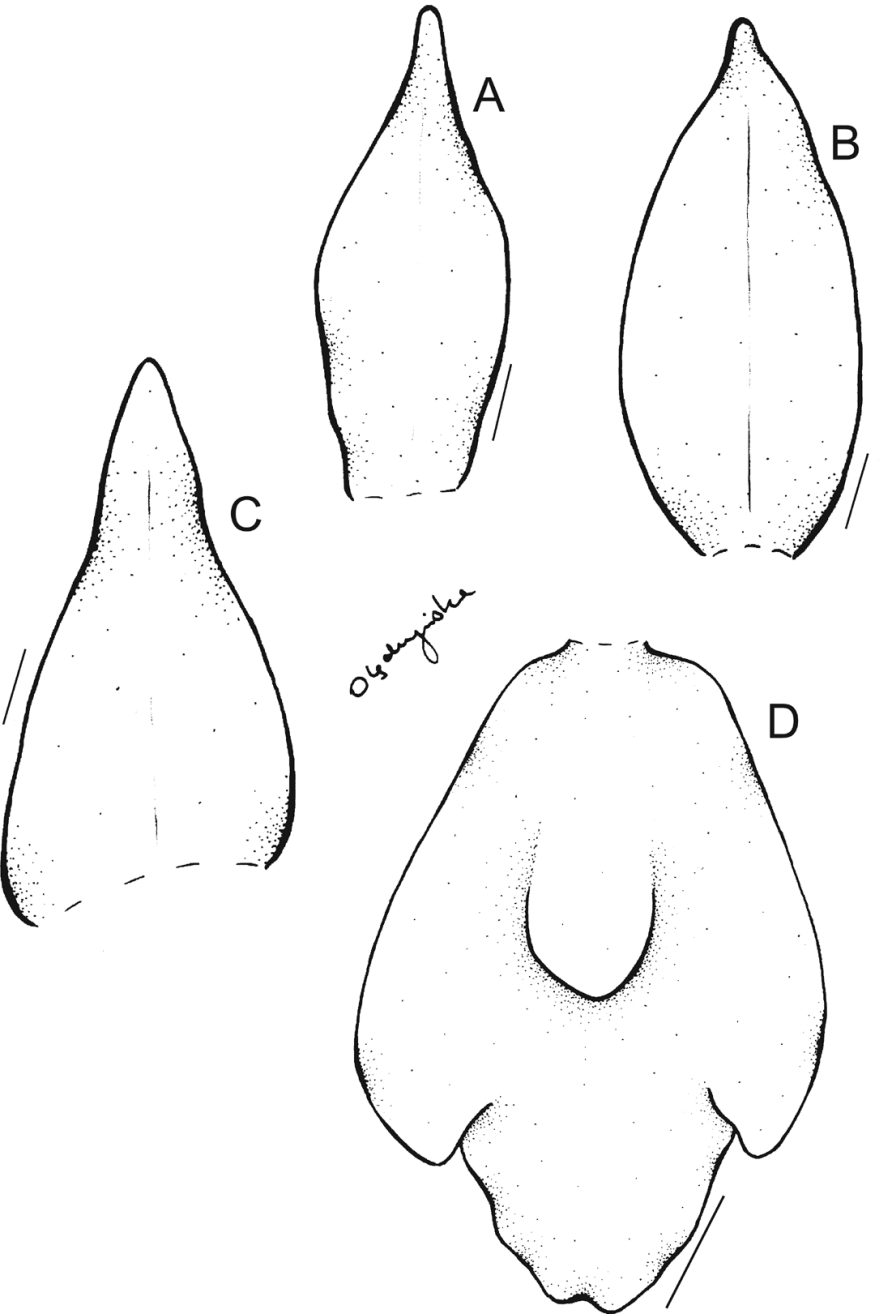
Drawing of the flower parts of *Maxillariasanderiana***A** petal **B** dorsal sepal **C** lateral sepal **D** lip. Scale bars: 10 mm. Redrawn from the type by N. Olędrzyńska.

**Figure 7.**
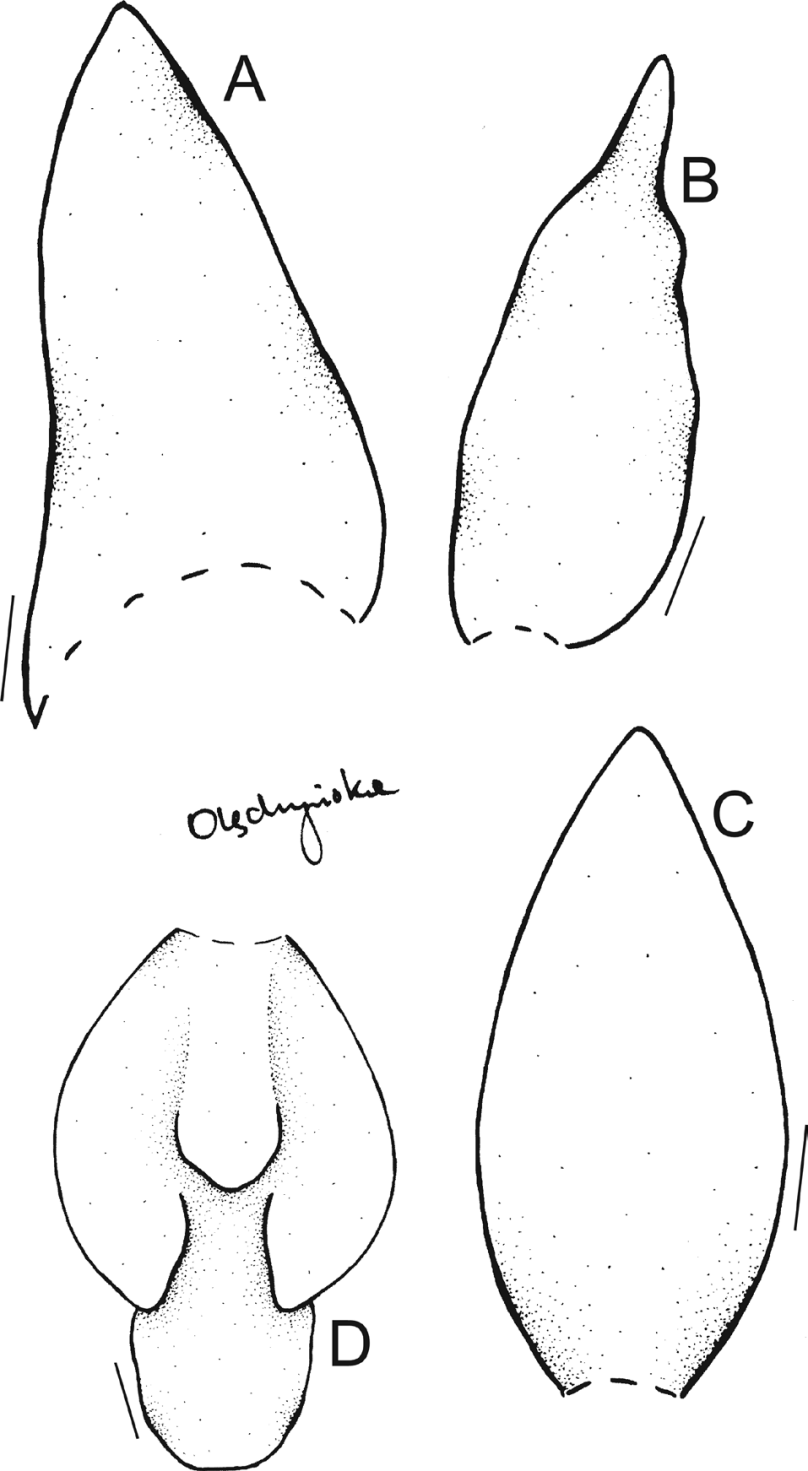
Drawing of the flower parts of *Maxillariagrandis***A** lateral sepal **B** petal **C** dorsal sepal **D** lip. Scale bars: 10 mm. Redrawn from the type by N. Olędrzyńska.

*Maxillariaanacatalinaportillae* is the only species morphologically similar to *M.sanderiana* (Fig. 6), *M.grandis* (Fig. 7), and *M.wojii* having flowers with yellow as basic color. Flowers of all aforementioned species are primarily white with various degrees of red-wine or maroon saturation.

The forms of the flower of *Maxillariasanderiana* and *M.anacatalinaportillae* are quite similar, but, again, the lip middle lobe of the former has strongly undulated and planar margins, and petals are shorter than dorsal sepal (vs equal in length in our new species). The micromorphology seems to be the same – in both cases, the lip surface is predominantly covered with pseudopollen, which is formed by the fragmentation of multicellular, moniliform trichomes (Davies et al. 2000). The populations of both species are isolated and are located at considerable distance from each other: *M.anacatalinaportillae* is known only from Maldonado (Carchi Province, Cantón Tulcán), whereas the closest population of *M.sanderiana* is located in the Baeza (Napo Province), ca. 400 km from Maldonado. *Maxillariawojii* can be easily distinguished from all other species mentioned above by the unique lip callus, consisting of the main part flanked by pair of subsidiary calli. Lip callus of *M.anacatalinaportillae* is very massive, flanked by narrow wings on each side, which seems to be an intermediate state between those found in *M.wojii* and *M.sanderiana*. The morphological differences and similarities have been summarized in Table 1.

**Table 1.** Summary of the morphological differences between the most similar species.

	*M.anacatalinaportillae*	*M.grandiflora*	*M.grandis*	*M.sanderiana*	*M.wojii*
Distribution	Ecuador	Colombia	Ecuador, Peru	Ecuador, Peru	Colombia
Habit	Caespitose epiphytes	Caespitose to ascending epiphytes	Caespitose epiphytes	Caespitose terrestrials or epiphytes	Caespitose epiphytes
Pseudobulbs	Ellipsoid to almost orbicular, compressed	Oblong-ellipsoid, compressed	Oblong-lanceolate, compressed	Ovoid, subglobose or oblong-ellipsoid, compressed	Elliptic, compressed
Leaves	Ligulate to oblong-elliptic, acute to shortly acuminate, petiolate	Lanceolate, acuminate, petiolate	Ligulate-lanceolate to oblong-lanceolate, acute, petiolate	Oblong-elliptic to broadly elliptic, acute, petiolate	Oblong-elliptic, acute, arching, petiolate
Flowers	Large and showy, scentless, campanulate, not fully opened, resupinated	Showy, triangular in outline, resupinated	Showy, large, resupinated	Large, showy, faintly fragrant during the day, variably marked, resupinated	Showy, wide-spreading, fleshy, resupinated
Dorsal sepal	Elliptic-ovate, concave along midvein, apex subobtuse, canaliculated	Elliptic, acute, rigid, concave, keeled along the back along the midvein	Oblong elliptic-ovate, acute, lightly concave	Elliptic-lanceolate to suborbicular. acuminate to obtuse-apiculate, lightly concave	Oblong-triangular. acute, keeled, with shallowly revolute lateral margins
Lateral sepals	Obliquely oblong triangular, somewhat concave at the base, apex, thickened, subobtuse	Triangular, acute-acuminate, strongly divergent, with minutely revolute lateral margins	Obliquely ovate-triangular, recurved or twisted near the middle	Obliquely ovate-triangular, recurved above the middle	Ovate-triangular, acute, with revolute lateral margins
Petals	Oblong lanceolate to ligulate-lanceolate, falcate at the base, apex attenuate, thickened, subobtuse	Elliptic-lanceolate, acute, indexed forming a chamber with the lip, with strongly recurved apices	Triangular with toothed margins, undulate and strongly recurved	Oblong-triangular to broadly ovate, abruptly acuminate	Oblong with an abruptly triangular apex, acuminate, recurved toward the apex
Lip	3-lobed, middle lobe broadly cordate or triangular when spread, concave along midvein, margins crenulate-undulate, fold-down, lateral lobes oblong-ovate in outline, canaliculated in natural position	Obscurely 3-lobed, strongly arched at the middle, lateral lobes rigidly erect, rounded, midlobe broadly ovate-triangular, obtuse, with undulate margins	Deeply 3-lobed, arching in natural position, lateral lobes obliquely elliptic, midlobe oblong-elliptic, obtuse, margins undulate and planar	3-lobed, arching, lateral lobes erect-incurved, obliquely obovate, midlobe ovate, obtuse, with undulate margins	3-lobed, arching, lateral lobes erect, transversely oblong, obtuse-rounded, midlobe ovate, obtuse, undulate-crenulate
Callus	Massive, reaching beyond the midpoint of lip, ligulate-ovate, flanked by narrow wing on each side	Ligulate, broad	Large, massive in the basal third of the lip	Large longitudinal, from the base of the lip to the middle, obtuse-rounded	Biseriate, central callus oblanceolate, obtuse, flanked at the apex by a pair of low, irregular, subsidiary calli
Column	Apically upcurved	Slightly curved	Arching	Arching	Arching

### ﻿Micromorphology

It is estimated that as many as 56% of the representatives of *Maxillariasensu lato* attract pollinators with “empty promises”, which are a combination of visual, tactile, and olfactory stimuli (Davies et al. 2005 and references therein). Among the species that offer any kind of reward, there are three types: nectar, pseudopollen (*farina*), and wax-like substances (Davies et al. 2003a, b, 2005). Until now, the micromorphology of only twelve members of *Maxillariagrandiflora*-complex has been studied. A common feature among its representatives is the presence of pseudopollen and at least 30 Ecuadorian species of *Maxillaria* produce pseudopollen (Davies et al. 2000). *M.anacatalinaportillae* is no exception and SEM analysis revealed the presence of copious moniliform trichomes and pseudopollen grains on the lip surface, mainly middle lobe and callus. Lip base and lateral lobes were rather smooth, with villiform to obpyriform papillae towards the middle part of the lip. (Davies and Turner 2004). Pseudopollen is usually produced by the fragmentation of labellar trichomes and it has the form of a whitish layer with a powder-like structure. It can be considered as a substitute reward for real pollen.

The presence of obpyriform and moniliform trichomes is typical for members of the *Maxillariagrandiflora*-complex (Davies and Turner 2004; Lipińska and Kowalkowska 2018) and is not surprising, since wherever pseudopollen-forming trichomes occur, labellar papillae tend to be obpyriform (Davies and Turner 2004).

The main pollinators of *Maxillaria* are stingless bees (Meliponini) (Roubik 2000). According to Davies et al. (2000), some of the members of the *Maxillariagrandiflora*-complex are pollinated by different insects: *Maxillariafletcheriana* Rolfe by the bumblebee *Bombusvolucelloides* Gribodo, *M.grandiflora* and *M.sanderiana* by *Eulaemacingulata* Fabricius. It is believed that bees collect pseudopollen from the flowers because of the nutrients it contains (Davies et al. 2000) and these include starch, oils, and proteins (van der Pijl and Dodson 1966). This may suggest that *M.anacatalinaportillae* is also pollinated by bees, similarly to closely related *M.grandiflora* and *M.sanderiana*.

## Supplementary Material

XML Treatment for Maxillaria anacatalinaportillae

## ﻿Appendix 1

**Table A1.** GenBank accession numbers: taxon, accession number for ITS and matK, respectively (asterisk states for sequences obtained in this research).

Species	ITS	*matK*
*Maxillariaangustissima*	DQ210512	DQ210961
*Maxillariaarachnitiflora*	DQ210242	DQ210758
*Maxillariaaugustaevictoriae*	DQ210027	DQ210599
*Maxillariaanacatalinaportillae* (*sp. nov.*)	OK032114*	OK032062*
*Maxillariacf.anatomorum*	DQ210483	DQ210966
*Maxillariacf.bennettii*	DQ210352	DQ210849
*Maxillariacf.Breviscapa*	DQ210544	DQ211019
*Maxillariacf.Grandiflora*	DQ210026	DQ210598
*Maxillariacf.Hillsii*	DQ210073	DQ210616
*Maxillariachionantha*	DQ210486	DQ210792
*Maxillariaconfusa*	DQ210513	DQ210840
*Maxillariacrocea*	DQ210311	DQ210634
*Maxillariaeburnea*	DQ210454	DQ210938
*Maxillariaecuadorensis*	DQ210508	DQ210771
*Maxillariaelegantula*	DQ210543	DQ210921
*Maxillariaexaltata*	DQ210320	DQ210818
*Maxillariafletcheriana*	DQ210209	DQ210739
*Maxillariafractiflexa*	DQ210074	DQ210617
*Maxillariagalantha*	DQ210574	DQ211049
*Maxillariagentryi*	DQ210492	DQ210845
*Maxillariagrandis*	DQ210368	DQ210862
*Maxillariaguadalupensis*	DQ210504	DQ210983
*Maxillariahuebschii*	OK032113*	OK032061*
*Maxillariairrorata*	DQ210430	DQ210917
*Maxillarialehmannii*	DQ210268	DQ210778
*Maxillarialepidota*	DQ210562	DQ210857
*Maxillarialongicaulis*	DQ210510	DQ210623
*Maxillarialongiloba*	DQ210432	DQ210919
*Maxillarialongipes*	DQ210519	DQ210999
*Maxillarialueri*	DQ210471	DQ210802
*Maxillariamelina*	OK030847*	OK032060*
*Maxillariameridensis*	DQ210427	DQ210780
*Maxillariamodesta*	DQ210195	DQ210726
*Maxillariamolitor*	DQ210370	DQ210863
*Maxillariamultiflora*	DQ210186	DQ210716
*Maxillariapachyacron*	DQ210489	DQ210593
*Maxillariapatens*	DQ210528	DQ210986
*Maxillariapauciflora*	DQ210390	DQ210631
*Maxillariaplatypetala*	DQ210558	DQ211033
*Maxillariaporrecta*	DQ210568	DQ210576
*Maxillariapseudoreichenheimiana*	DQ210328	DQ210827
*Maxillariaramonensis*	DQ210099	DQ209918
*Maxillariareichenheimiana*	DQ210503	DQ210827
*Maxillariarobusta*	DQ210192	DQ210722
*Maxillariarotundilabia*	DQ461792	DQ210893
*Maxillariasanderiana*	DQ210271	DQ210781
*Maxillariasanderiana*	DQ210453	DQ209967
*Maxillariasetigera*	DQ210230	DQ210674
*Maxillariasilvana*	DQ210516	DQ210997
*Maxillariaspeciosa*	DQ210075	Q210618
*Maxillariastriata*	DQ210267	DQ210777
*Maxillariaturkeliae*	DQ210276	DQ209945
*Trigonidiumobtusum*	DQ210220	DQ210641
